# Regulation of antiviral immune response by African swine fever virus (ASFV)

**DOI:** 10.1016/j.virs.2022.03.006

**Published:** 2022-03-09

**Authors:** Xiaojie Zheng, Shengming Nie, Wen-Hai Feng

**Affiliations:** a State Key Laboratory of Agrobiotechnology, College of Biological Sciences, China Agricultural University, Beijing, 100193, China; b Ministry of Agriculture Key Laboratory of Soil Microbiology, College of Biological Sciences, China Agricultural University, Beijing, 100193, China; c Department of Microbiology and Immunology, College of Biological Sciences, China Agricultural University, Beijing, 100193, China

**Keywords:** African swine fever virus (ASFV), Innate immunity, Adaptive immunity, Immune evasion, Vaccine

## Abstract

African swine fever (ASF) is a highly contagious and acute hemorrhagic viral disease with a high mortality approaching 100% in domestic pigs. ASF is an endemic in countries in sub-Saharan Africa. Now, it has been spreading to many countries, especially in Asia and Europe. Due to the fact that there is no commercial vaccine available for ASF to provide sustainable prevention, the disease has spread rapidly worldwide and caused great economic losses in swine industry. The knowledge gap of ASF virus (ASFV) pathogenesis and immune evasion is the main factor to limit the development of safe and effective ASF vaccines. Here, we will summarize the molecular mechanisms of how ASFV interferes with the host innate and adaptive immune responses. An in-depth understanding of ASFV immune evasion strategies will provide us with rational design of ASF vaccines.

## Highlights

• We have summarized current progresses on how ASFV affects host immune responses.
• Understanding virus-host interaction is essential for us to elucidate ASFV pathogenesis.
• We have discussed ASFV proteins that can suppress host immune responses.
• This review will provide us with some clues on rational design of ASFV vaccines.

## 1 Introduction

African swine fever (ASF) is a highly contagious viral disease with a mortality rate approaching 100%, leading to significant economic losses in swine industry worldwide (Wang et al., 2019). ASF was first discovered in Kenya in 1921. It occurs in an ancient sylvatic cycle with warthog (Montgomery, 1921). The causative agent of ASF is African swine fever virus (ASFV), a large double-stranded enveloped DNA virus. It is the sole member of the family *Asfarviridae* and belongs to nucleocytoplasmic large DNA viruses (NCLDVs) (Dixon et al., 2019). The ASFV particle possesses a multilayered structure with an overall icosahedral morphology and a diameter of 260–300 ​nm (Wang et al., 2019). ASFV genome is approximately 170–193 ​kb in length and codes for 150–167 proteins, depending on the virus strains (Sanchez-Vizcaino et al., 2015; Simoes et al., 2019). These proteins dedicate not only to virus replication but also to the evasion of host defenses. Until now, about half of ASFV genes lack any known or predictable functions (Dixon et al., 2017; Alejo et al., 2018). ASFVs are classified into 24 different genotypes based on the 3′- end sequences of the *B646L* gene, which encodes the major capsid protein p72 (Muangkram et al., 2015).

In the 1950s, ASFV rapidly spread throughout Europe and South America, where it was eradicated by strict control and effective eradication programs except on the island of Sardinia in the mid-1990s (Iglesias et al., 2017). ASFV was introduced into Georgia in 2007, and then spread to EU countries including Russia (2007), Ukraine (2012), Belarus (2013), Poland (2014), Lithuania (2014), Latvia (2014), Estonia (2014), Romania (2017), Czech Republic (2017) and Hungary (2018) (Zhao et al., 2019). On August 3, 2018, a new ASF epidemic was reported in Liaoning Province of China. By February 12, 2021, more than 183 ASF cases (including four outbreaks in wild boars) have occurred in 31 provinces or regions in China, of which at least 130 cases have a mortality rate of more than 50% or even 100%. Different from the past Georgia-07-like genotype II ASFV, the main epidemic strain in China, two genotype I ASFVs, HeN/ZZ-P1/21 and SD/DY-I/21, are recently isolated from pig farms. Animal challenge testing reveals that SD/DY-I/21 exhibits low virulence and high transmissibility in pigs, which causes more difficulties and challenges for the early diagnosis and control of ASF (Sun et al., 2021). Now, ASF has posted a great challenge to our government to protect the swine industry.

The natural reservoir hosts of ASFV are the warthogs (*Phacochoerus africanus*) and bush pigs (*Potamchoerus larvatus*). Soft ticks of the *Ornithodoros* species can be infected over long time periods and act as virus reservoirs (Dixon et al., 2019). Recently, accumulating evidence has suggested that leeches can serve as another possible reservoir for ASFV (Karalyan et al., 2019). ASFV mainly infects mononuclear phagocytes of the myeloid lineage, including monocytes, macrophages and dendritic cells (DC), by two alternative endocytic mechanisms: clathrin-mediated endocytosis and macropinocytosis (Hernaez et al., 2016; Franzoni et al., 2018). CD163 has been found to help ASFV infect host cells, but this observation is debatable (Sanchez-Torres et al., 2003; Popescu et al., 2017).

With the high development of globalization, it is difficult to eliminate ASFV by traditional ways, such as culling and movement restriction. In order to effectively eradicate the potential threat of ASFV, a safe and effective vaccine is an ideal choice. Although ASFV has been around for a century, there is still no licensed vaccine. The main factor limiting the development of safe and effective ASF vaccines is the insufficient understanding of ASFV pathogenesis and how ASFV evades host immune response.

Recently, there are increasing reports demonstrating that ASFV has evolved various mechanisms to antagonize host immune responses for efficient infection and cause extremely high mortality (Razzuoli et al., 2020; Wang J. et al., 2020; Wang T. et al., 2020). In this review, we will focus on the interaction between host immune system and ASFV, describing the immunoevasion strategies adopted by ASFV and its proteins to modulate host immune responses and signaling pathways, and discussing the implications that these modulations have on viral virulence. The immune escape strategies, including inhibition of interferons production or functions, modulation of inflammatory responses, regulation of apoptosis, and suppression of adaptive immune response, are summarized.

## 2 Escape from innate immunity

### 2.1 Inhibition of interferon (IFN) production or functions

**Fig. 1:**
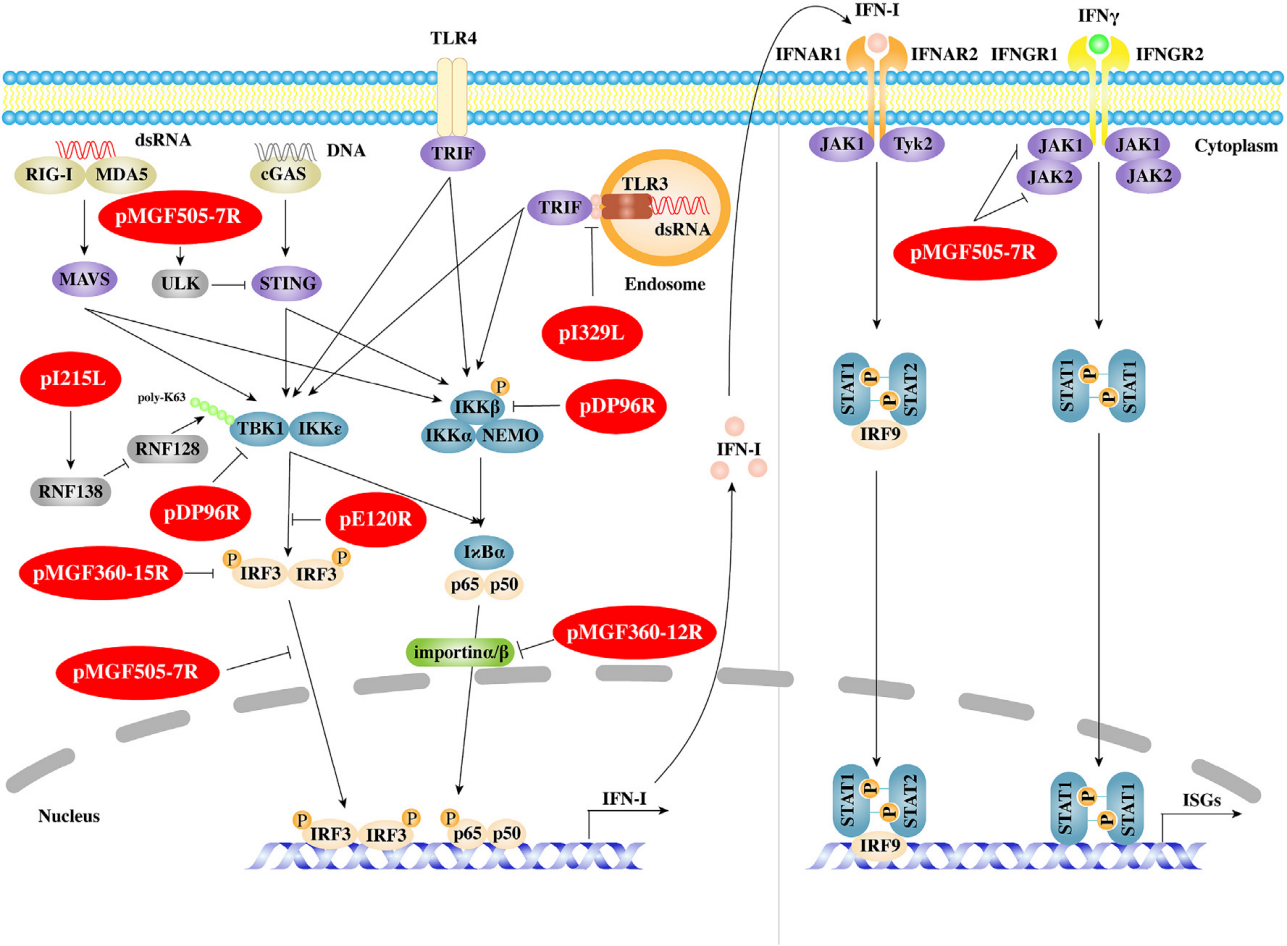
Inhibition of IFN production or functions by African swine fever virus (ASFV). ASFV proteins are shown in red ellipses. ASFV pMGF360-12L competitively binds nuclear transport protein KPNA2, KPNA3 and KPNA4 with NF-κB, thereby inhibiting NF-κB nuclear translocation and IFN-β production. pMGF360-15R (pA276R) suppresses IFN-β expression by targeting IRF3. pI329L competes with TLR3 for adaptor protein TRIF to inhibit IFN-I production. pDP96R affects cGAS/STING-mediated NF-κB signaling by blocking the activation of TBK1 and IKKβ. pE120R interacts with IRF3 and interferes with the recruitment of IRF3 to TBK1, which in turn suppresses IRF3 phosphorylation to decrease IFN-β production. pI215L interacts with E3 ubiquitin ligase RNF138 and promotes RNF138 to degrade RNF128, which results in reduced K63-linked polyubiquitination of TBK1 and IFNβ production. pMGF505-7R facilitates STING degradation by promoting the expression of ULK1 and blocks IRF3 nuclear translocation to negatively regulate cGAS-STING signaling pathway. In addition, pMGF505-7R also inhibits IFN-γ signaling pathway by suppressing JAK1 and JAK2.

IFNs have great differences in structure, receptor distribution and tissue-specific biological activities, but all can induce an antiviral state (van Boxel-Dezaire et al., 2006). In particular, type I IFNs (IFN-I) represent one of the first lines of defense to limit viral replication and spread by inducing multiple antiviral proteins that interfere with every step of the viral life cycle, thus contributing to the protection of hosts from infections. When a virus infects hosts, various pattern recognition receptors (PRRs), including Toll-like receptors (TLRs), RIG-I-like receptors (RLRs) and cytoplasmic DNA sensors, recognize pathogen-associated molecular patterns (PAMPs) and result in the activation of innate immune signaling pathways to produce proinflammatory cytokines and IFN-I (Zhu et al., 2019). Viruses, which have coevolved with their hosts, develop strategies to counteract the signaling cascades of the IFNs system and ensure their replication. Recent studies have shown that virulent strains of ASFV can suppress the expression of IFNs and IFN-stimulated genes (ISGs) in infected cells (Garcia-Belmonte et al., 2019; Razzuoli et al., 2020; Li D. et al., 2021a). Of ASFV proteins, multigene family 360 (MGF360), MGF530/505, pI329L, pDP96R, pE120R, and pI215L are demonstrated to inhibit IFN-I responses (Fig. 1).

#### 2.1.1 MGF 360 and MGF 530/505

ASFV MGFs, including MGF100, MGF110, MGF300, MGF360, and MGF505/530, are located within the left 40 ​kb and right 20 ​kb of the genome (Dixon et al., 2013). Among these MGFs, MGF360, containing 11 to 15 members, and MGF530/505, containing 9 or 10 members, are shown to suppress IFN-I responses and improve the proliferation efficiency of virus by prolonging the survival time of infected cells (Afonso et al., 2004; O'Donnell et al., 2015; Reis et al., 2016).

NF-κB functions as a nuclear transcription factor that regulates the expression of genes influencing a broad range of biological processes, including immunity and inflammation. It is shown that activated NF-κB nuclear translocation requires the assistance of karyopherins, such as importin α (KPNA1-6) and importin β (KPNB1), which contain a nuclear localization signal (NLS) (Stewart, 2007; Beaudet et al., 2020). pMGF360-12L, as a member of MGF360, has 350 amino acids, of which 53 amino acids at the N-terminal are predicted to have nuclear localization signals. It is demonstrated that pMGF360-12L significantly inhibits IFN-β signaling pathway and reduces IRF3, AP-1, NF-κB, and TBK1 expression following poly(I:C) or TNFα stimulation. Further research shows that pMGF360-12L competitively combines KPNA2, KPNA3 and KPNA4 with NF-κB in a NLS dependent manner, thereby inhibiting NF-κB nuclear translocation and host antiviral response (Zhuo et al., 2021). Importantly, deletion of *MGF360-12L* from Benin 97/1 strain can lead to a higher level of IFN-I production in porcine alveolar macrophages (PAMs) (Reis et al., 2016).

Some other MGF360 family proteins with unclear mechanisms can also suppress the production or function of IFNs. For instance, pA276R/pMGF360-15R is able to regulate the expression of IFN-β by targeting IRF3 in an NF-κB independent manner, but does not affect the JAK-STAT pathway activation by either type I or type Ⅱ IFN (Correia et al., 2013). However, recombinant NH/P68 virus strain lacking *A276R* (NH/P68 ΔA276R) shows the same infectivity as the parental strain *in vitro* and fails to protect pigs from genotype II Armenia07 strain challenge (Gallardo et al., 2018).

The cGAS-STING pathway is important for IFNs production during DNA virus infection (Tan et al., 2018). cGAS senses cytosolic dsDNA and produces a cyclic dinucleotide, 2′3′-cGAMP, to activate the endoplasmic reticulum (ER)-localized protein STING. Then STING activates IRF3 and NF-κB transcription factors to induce the expressions of IFN-I and inflammatory cytokines. pMGF505-7R/pA528R, as a member of MGF505/530, has evolved not only to inhibit poly(I:C)-mediated activation of IRF3 and NF-κB, but also to suppress the response of either type I and type Ⅱ IFN (Correia et al., 2013). Recently, it has been reported that pMGF505-7R also inhibits cGAS-STING pathway mediated IFN-I expression. pMGF505-7R interacts with both STING and ULK1, leading to the phosphorylation of STING on S366 by ULK1 and STING degradation via autophagy pathway (Li D. et al., 2021b). Deletion of *MGF505-7R* from ASFV CN/GS/2018 strain induces more serum IFN-β and lower virus replication in blood and tissues of infected pigs in comparison with parental ASFV. Moreover, ASFV HLJ/18 strain pMGF505-7R inhibits IFN-I production by directly interacting with IRF3 and blocking its nuclear translocation (Li J. et al., 2021).

Previously, it was reported that pMGF505-7R reduced the response of type Ⅱ IFN, but its mechanism was not clear (Correia et al., 2013). A recent report demonstrates that pMGF505-7R inhibits IFN-γ mediated transcription of downstream genes, and interacts with JAK1 and JAK2 to impair IFN-γ signaling pathway (Li D. et al., 2021a). Further researches show that pMGF505-7R interacts with E3 ubiquitin ligase RNF125 to promote its expression, leading to the degradation of JAK1. Meanwhile, pMGF505-7R interacts with transcription factor Hes5 to inhibit its expression, causing the downregulation of JAK2. Deletion of *MGF505-7R* from ASFV CN/GS/2018 strain is verified to enhance the JAK-STAT1 signaling pathway compared to its parental virus in PAMs. ASFV-ΔMGF505-7R is attenuated and induces more serum CXCL9 in pigs (Li D. et al., 2021a). These results imply that MGFs have the ability to inhibit IFNs production and function.

#### 2.1.2 pI329L, a viral toll-like receptor (TLR) homologue

pI329L is a late protein of ASFV, which contains 329 amino acids and its sequence is relatively conservative. It has been shown that pI329L is a highly glycosylated protein distributed on the membrane of infected cells, with a signal peptide (amino acids 1–17), an N-terminal extracellular domain (amino acids 18–239), a transmembrane domain (amino acids 240–260), and a C-terminal intracellular domains (amino acids 261–329). Through multiple alignments with Toll-like receptor (TLR) proteins, it shows that the intracellular domain of pI329L is similar to BOX1 and BOX2 regions of human Toll-interleukin-1 receptor (TIR) like domain of TLR3, with 35% sequence similarity. Importantly, the extracellular domain of pI329L contains four leucine-rich repeats (LRR) (de Oliveira et al., 2011). LRR exists in several TLRs and is an important motif for protein-protein interactions (Mukherjee et al., 2019). These results suggest that pI329L may competitively bind to the downstream signal molecules of TLRs, thus affecting IFNs production.

As previously studied, pI329L, a viral TLR3 antagonist, inhibits the activation of IFN-I stimulated by dsRNA or LPS. Based on structural analysis, pI329L is predicted to target TRIF, an adaptor protein in the TLR3 pathway. Overexpression of TRIF eliminates the influence of pI329L on the activation of IRF3 and NF-κB and the expression of IFNs (de Oliveira et al., 2011; Henriques et al., 2011). Meanwhile, attenuated ASFV OURT88/3 strain lacking *I329L* increases IFN-I expression compared with its parental virus in infected macrophages, but remarkedly reduces protection against challenges with the virulent ASFV OURT88/1 strain in pigs. Unexpectedly, deletion of *I329L* does not attenuate ASFV Georgia 2007/1 strain (Reis et al., 2020).

#### 2.1.3 pDP96R, a virulence factor

DP96R is present in the right variable region of ASFV genome, and is an early expressed protein with a molecular weight of ∼10.7 ​kDa. Its amino acid sequence is highly conserved among diverse ASFV strains. Previous studies have indicated that the recombinant virus, with *DP96R* gene deletion (ΔUK) from ASFV E70 strain, has no changes in replication *in vitro*, but exhibits decreased virulence in infected pigs (Zsak et al., 1998). Interestingly, a recent study shows that Georgia 2007/1 mutant (ASFV-G-Δ9GL/ΔUK) with double gene 9GL (B119L) and UK (DP96R) deletions not only affects virus replication in cells, but also reduces virus pathogenicity in domestic pigs (O'Donnell et al., 2017). Clearly, these data fully support that DP96R is a virulence factor of ASFV.

ASFV has been confirmed to activate and regulate cGAS-STING signaling pathways (Garcia-Belmonte et al., 2019). There is a report indicating that pDP96R negatively regulates IFN-I production via cGAS-STING signaling pathway and affects NF-κB signaling by blocking the activation of TBK1 and IKKβ (Wang et al., 2018). These data suggest that pDP96R plays an important role in ASFV immune evasion.

#### 2.1.4 pE120R, a viral transport associated protein

pE120R is a capsid component associated with the major capsid protein p72. It is highly conserved among different ASFV strains and contains approximately 120 amino acids (Liu et al., 2021). There is a report showing that pE120R is essential for the microtubule-mediated transport of virus particles from virus factories to the plasma membrane but is not required for the assembly of morphologically mature virions in ASFV BA71V strain (Andres et al., 2001). ASFV CN/GS/2018 strain (a genotype II ASFV) lacking *E120R* couldn't be rescued in porcine BMDM cells (Liu et al., 2021). It has been described that pE120R is post-translationally acetylated at the N-terminal alanine (Ala) residue, which might be relevant to ASFV life cycle (Alfonso et al., 2007). Besides, purified pE120R interacts with DNA in a sequence-independent manner, indicating a possible role of pE120R in the encapsidation of ASFV DNA (Martinez-Pomares et al., 1997; Alfonso et al., 2007).

It is not until 2021 that pE120R is found to play a role in ASFV immune escape by suppressing cGAS-STING-triggered IFN-β production. Detailed analysis reveals that pE120R interacts with IRF3 carboxyl terminal domain (CTD) to interfere with the recruitment of IRF3 to TBK1, which in turn suppresses IRF3 phosphorylation and subsequently decreases IFN-β production. Additionally, the 72 and 73 aa sites of pE120R are essential for its inhibitory activity. E120R Δ72–73aa recombinant virus abrogates the interaction between IRF3 and pE120R, enhances IFN-β and numerous ISGs expression, and suppresses ASFV replication compared to wild type ASFV in PAMs (Liu et al., 2021).

#### 2.1.5 pI215L, a viral E2 ubiquitin-conjugating enzyme

pI215L, a very early protein of ASFV, shuttles between the nucleus and cytoplasm and can be found in viral factories (Bulimo et al., 2000; Freitas et al., 2018). pI215L is the only known E2-ubiquitin conjugating enzyme encoded by the virus. As previously described, pI215L has 31%–45% identical amino acids in the conserved N-terminal region and 52%–66% similarity when compared with other ubiquitin-conjugating (UBC) enzymes (Hingamp et al., 1992). This viral E2-ubiquitin conjugating enzyme can ubiquitinate some ASFV proteins such as PIG1 to induce limiting proteolysis (Hingamp et al., 1995). It has been shown that pI215L is essential for ASFV genome replication, viral late gene transcription and progeny production (Freitas et al., 2018). Further studies show that pI215L interacts with 40S ribosomal protein RPS23, the cap-dependent translation initiation factor eIF4E, and the E3 ubiquitin ligase Cullin 4B to alter the mTOR signaling pathway and impact the host translation machinery (Barrado-Gil et al., 2020). Additionally, pI215L also binds to SMCp, which contains an A/T rich interaction domain (ARID) DNA binding domain. However, its significance is unknown (Bulimo et al., 2000).

A recent report has identified that pI215L is a strong inhibitor of IFN-I. Knockdown of pI215L inhibits ASFV HLJ/18 strain replication and enhances IFN-β production (Huang et al., 2021). Further research shows that pI215L suppresses K63-linked polyubiquitination of TBK1, an important post-translational modification of TBK1 activation, and thus inhibits IFN-β production. However, this is independent of its E2 ubiquitin conjugating enzyme activity. pI215L interacts with E3 ubiquitin ligase RNF138 to enhance the interaction between RNF138 and another E3 ubiquitin ligase RNF128, resulting in the degradation of RNF128 and reduced K63-linked polyubiquitination of TBK1 (Huang et al., 2021).

### 2.2 Regulation of inflammatory responses

**Fig. 2:**
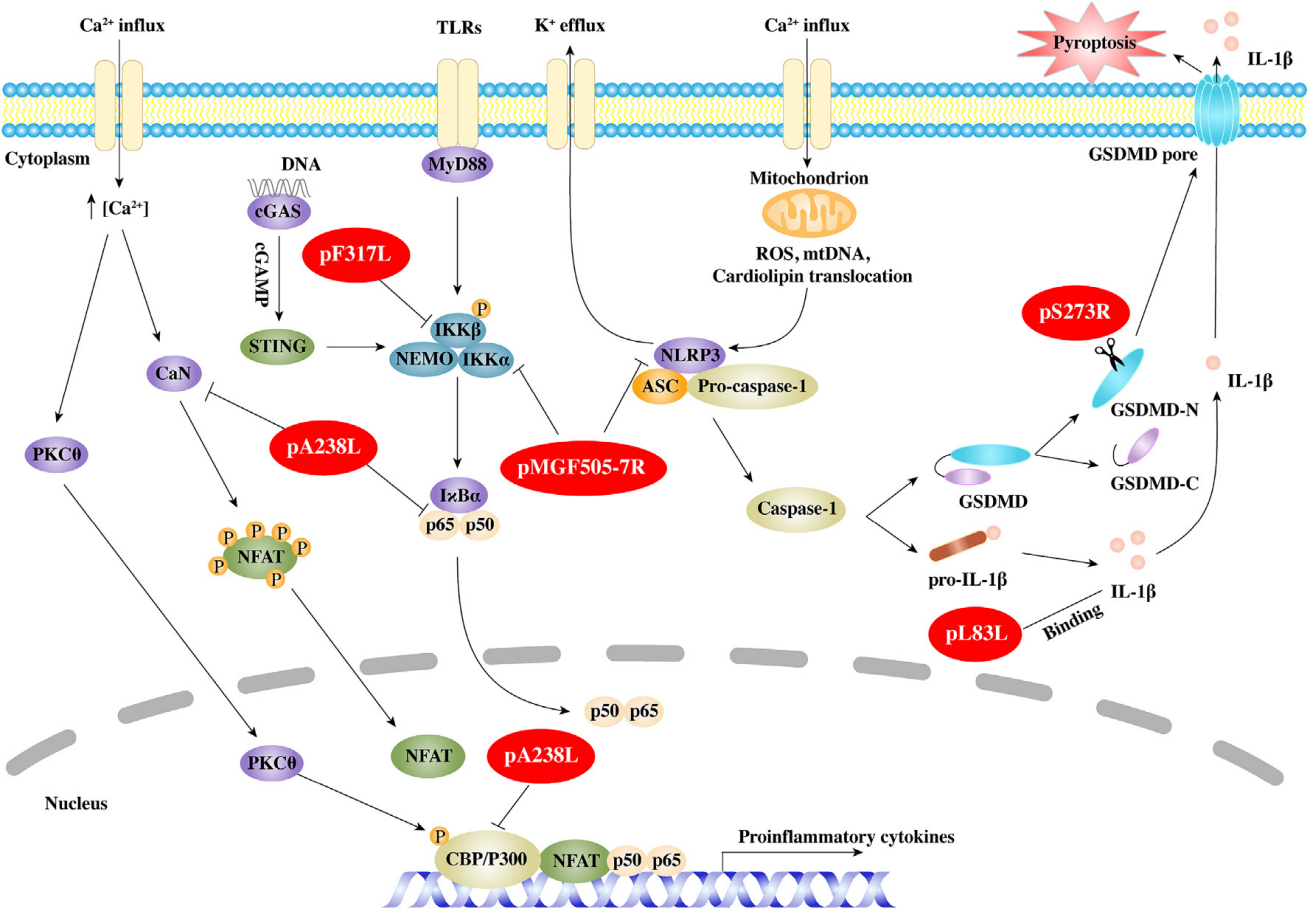
Regulation of inflammatory responses by African swine fever virus (ASFV). ASFV proteins are shown in red ellipses. ASFV pA238L, an IκB homolog protein, inhibits the activation of host transcription factors, including NFAT, NF-κB, and CBP/p300, to regulate virous proinflammation cytokines. pMGF505-7R (pA528R) not only interacts with IKKα to inhibit IκBα phosphorylation and NF-κB nuclear translocation, but also interacts with NLRP3 to suppress mature IL-1β production. pL83L, a putative IL-1β binding protein. pF317L interacts with IKKβ and inhibits IKKβ phosphorylation leading to suppress the activation of NF-κB. pS273R cleaves GSDMD at G107–A108 to produce a shorter N-terminal fragment of GSDMD (GSDMD-_N1–107_), which is unable to induce pyroptosis.

The inflammatory response induced by ASFV plays an important role in the pathogenesis of ASFV. It is reported that ASFV induces macrophages to release TNFα, IL-1β, IL-6, IL-8 and other inflammatory cytokines (Zhang et al., 2006). Furthermore, the levels of TNFα, IL-12p40, IL-23, IL-17, and G-CSF in serums of ASFV-infected pigs are significantly increased (Zakaryan et al., 2015; Karalyan et al., 2016). At present, it is generally believed that ASFV can induce a strong inflammatory response, leading to serious inflammatory lesions and death of infected pigs. However, there are a large number of studies showing that the level of inflammatory factors induced by low virulence ASFV strains is significantly higher than that induced by high virulence strains. For example, the expression of IFN-α, TNFα, IL-12p40 induced by the low virulent strain ASFV/NH/P68 is higher than that induced by the high virulent strain ASFV/L60 in macrophages (Gil et al., 2008). Compared with the high virulent strain ASFV Benin97/1, the low virulent strain ASFV/OURT88/3 induces macrophages to produce higher levels of IL-1α, IL-1β, IL-18, CCL4, CXCL10 (Fishbourne et al., 2013; Franzoni et al., 2018). These results imply that the high virulent ASFV strains can modulate the production of pro-inflammatory factors to evade host immune response. Several ASFV proteins, including pA238L, pMGF505-7R, pL83L and pF317L and pS273R, have been confirmed to inhibit inflammatory responses and their mechanisms of action will be described in details below (Fig. 2). In addition to these proteins, a recent paper has shown that pS183L, pE199L, pO61R and pI7L activate the inflammatory response, whereas pI226L, pA151R, pNP419L and pQP383R inhibit the inflammatory response (Song et al., 2020).

#### 2.2.1 pA238L, an IκB homolog protein

pA238L, an early protein of ASFV, is present in both the nucleus and the cytoplasm in ASFV-infected cells. Two different forms of the A238L protein with molecular weight of 28 and 32 ​kDa have been found, which are considered to be the result of post-translational modification. However, the nature of the modification has not been determined (Silk et al., 2007). Previous studies have identified that pA238L, which contains ankyrin repeats in center of the protein, is 40% homology to porcine IκB, the inhibitor of NF-κB. By analyzing a NF-κB-dependent luciferase reporter gene in cells ectopically expressed pA238L, it is demonstrated that pA238L inhibits the expression of NF-κB controlled genes (Powell et al., 1996). Interestingly, only the higher 32 ​kDa form of pA238L co-precipitates with p65 but not p50, implying that p50/p65 heterodimers rather than p50/p50 homodimers is disturbed by pA238L (Tait et al., 2000). pA238L does not inhibit nuclear import or export of p65 (Silk et al., 2007).

It is shown that pA238L interacts with the catalytic subunit of the serine threonine protein phosphatase calcineurin (CaN) to affect its phosphatase activity (Miskin et al., 2000). The PxIxITxC/S motif, which is located in the C-terminus of pA238L downstream from the ankyrin repeats, is necessary for pA238L to bind CaN. CaN participates in a number of different pathways, including the activation of transcription factors of the NFAT family, to induce several cytokines such as IL-2, IL-4, and GM-CSF (Hogan, 2017). pA238L interacts with CaN to affect its phosphatase activity, thus inhibiting the activation of NFAT (Miskin et al., 2000). It has also been shown that pA238L suppresses the transcription of COX-2, which induces a strong lipid mediator of inflammation, in a NFAT-dependent manner, but not NF-κB pathway (Granja et al., 2004).

In addition, pA238L interacts with CBP/p300 in the nucleus to impair the recruitment of p300 to the transcription complex. It also disturbs the phosphorylation of p300 by PKC-θ to inhibit the p300 activity. As a consequence of the blockage, the expression of TNFα and iNOS are strongly inhibited (Granja et al., 2006a, 2006b). However, ASFV *A238L* deletion mutant from the highly virulent Malawi Lil-20/1 strain or E70 strain does not affect the viral pathogenicity in domestic pigs although the level of TNFα is increased (Neilan et al., 1997; Salguero et al., 2008).

#### 2.2.2 pMGF505-7R, a multifunctional protein

IL-1β is an effective multipotent proinflammatory cytokine produced mainly by monocytes, macrophages and lymphocytes, and plays a key role in regulating innate immune responses and adaptive immune responses (Aarreberg et al., 2018). The production and secretion of mature IL-1β requires two processes. The first is that PRRs sense the invasion of pathogenic microorganisms and promote the transcription and translation of IL-1β precursors (pro-IL-1β) through a variety of signal pathways, while the second is that inflammasome assembles and activates the caspase family proteins to cleaves pro-IL-1β to mature IL-1β (Takeuchi and Akira, 2010). Using specific siRNA targeting TLRs, NLRP3, or MyD88, ASFV is found to promote mature IL-1β production via TLRs/MyD88 pathway and NLRP3 inflammasome both *in vitro* and *in vivo*, but at low levels. Further exploration suggests that ASFV strongly inhibits inducer-mediated IL-1β, which might be responsible for why ASFV infection induces low levels of IL-1β (Li J. et al., 2021).

Through screening ASFV proteins, pMGF505-7R is found to have a strong ability to inhibit inducer-mediated IL-1β production. pMGF505-7R is shown to interact with IKKα to inhibit IκBα phosphorylation and NF-κB nuclear translocation, thereby suppressing transcription of pro-IL-1β. pMGF505-7R also interacts with NLRP3 to block NLRP3 inflammasome assembly to suppress mature IL-1β secretion (Li J. et al., 2021). Importantly, Deletion of *MGF505-7R* from ASFV HLJ/18 strain attenuates ASFV virulence and induces more IL-1β production compared with its parental ASFV strain *in vivo*.

#### 2.2.3 pL83L, a putative IL-1β binding protein

pL83L is encoded by *L83L* gene, a highly conserved protein across most ASFV strains, and predicted to be a prenylated protein (Marakasova et al., 2017). As an early expression protein, pL83L is a no-essential protein for virus replication. It is shown that pL83L specifically binds IL-1β via yeast two hybrid assay, suggesting that pL83L might inhibit the antiviral ability of IL-1β (Borca et al., 2018). However, deletion of *L83L* gene does not significantly change the pathogenicity of ASFV Georgia (ASFV-G) strain both *in vitro* and *in vivo* (Borca et al., 2018).

#### 2.2.4 pF317L, an uncharacterized protein

pF317L protein is an uncharacterized protein of ASFV. pF317L consists of 317 amino acids, and its function is still unknown (Dixon et al., 2013). A recent report shows that pF317L is an inhibitor of the inflammatory responses (Yang et al., 2021). pF317L inhibits TNFα and poly(dA:dT)-induced proinflammatory cytokine expression and NF-κB promoter activation. Further research proves that pF317L interacts with IKKβ and inhibits IKKβ phosphorylation, leading to decreased IκBα phosphorylation and ubiquitination as well as IκBα upregulation. Naturally, p317L also inhibits p65 nuclear translocation. It is found that the amino acids from 109 to 208 in pF317L are critical for its interaction with IKKβ and the suppression of NF-κB activation (Yang et al., 2021). Importantly, deletion of *F317L* from ASFV is lethal to the virus. However, ectopic expression of pF317L significantly promotes ASFV replication in iPAMs, and knockdown of *F317L* by specific siRNA clearly decreases ASFV replication (Yang et al., 2021). Nevertheless, whether knockdown of *F317L* has an effect on inflammatory response needs to be verified.

#### 2.2.5 pS273R, a SUMO-1-specific protease

Pyroptosis is critical for host to eliminate pathogen and engage inflammatory responses to potentiate protective host immunity (Kesavardhana et al., 2020). It has been reported that pyroptosis is activated by various bacterial and viral infection. In canonical pathway, after sensing PAMPs or danger signals, inflammasome sensors (such as NLRP1, NLRP3, NLRC4, AIM2 and pyrin) are assembled into heterologous multiprotein complexes known as inflammasome. The inflammasome acts as a scaffold to recruit and promote caspase-1 auto-processing activation. Activated caspase-1 not only cleaves pro-IL-1β or pro-IL-18 to mature IL-1β or IL-18, but also cleaves and activates gasdermin D (GSDMD). In non-canonical pathway, caspase-4/5/11 directly recognize cytosolic LPS to activated GSDMD (Broz and Dixit, 2016; Frank and Vince, 2019). Cleavage of GSDMD by caspases separates into a N-terminal fragment (31 ​kDa) and a C-terminal fragment (22 ​kDa). N-terminal fragment of GSDMD associates with the cell membrane to assemble into pores of 10–33 ​nm in diameter, leading to cell swelling and release of activated IL-1β and IL-18 (Chen et al., 2016; Ding et al., 2016; Liu et al., 2016).

pS273R is expressed at late stage of ASFV infection and localized in cytoplasmic viral factories. It has been proved that pS273R, containing 273-amino-acid, is a cysteine proteinase that belongs to SUMO-1-specific protease family (Andrés et al., 2001). pS273R protease contains a N-terminal “arm domain” and a C-terminal “core domain”. The “core domain” shares similar structure with other SUMO proteases and the “arm domain” is assumed to recruit its substrate proteins (Li et al., 2020). Early reports have shown that ASFV polyprotein precursor pp220 and pp62 are cleaved by pS273R to produce p15, p35, and p8 (from pp62) and p5, p34, p14, p37, and p150 (from pp220), which jointly participating in the assembly of ASFV core-shell (Andrés et al., 2001, 2002; Alejo et al., 2018). Thus, effective inhibition of pS273R affects ASFV particle maturation and infectivity (Alejo et al., 2003). A recent article shows that pS273R inhibits ASFV infection-induced pyroptosis to regulate inflammatory responses (Zhao et al., 2021). pS273R interacts with GSDMD and then cleaves GSDMD at G107–A108 to produce a shorter N-terminal fragment of GSDMD (GSDMD-N_1–107_). Unlike the canonical GSDMD-N_1–279_ produced by caspase-1, the GSDMD-N_1–107_ is unable to induce pyroptosis. Notably, pS273R further cleaves GSDMD-N_1-279_ to produce GSDMD-N_1-107_, which suppresses GSDMD-N_1-279_-induced pyroptosis to promote ASFV replication (Zhao et al., 2021).

### 2.3 Modulation of apoptosis

Apoptosis is a process of programmed cell death in multicellular organisms. It has been reported that apoptosis plays an important role in the pathogenesis of various virus infections, which is considered as an important natural defense mechanism for hosts to inhibit virus replication and eliminate virus infected cells. As a result, numerous viruses have evolved strategies to prevent or delay apoptosis during replication, ensuring cell survival until sufficient offspring viruses are produced (Chen and Lin, 2017; D'Arcy, 2019; Fan, 2019).

**Fig. 3:**
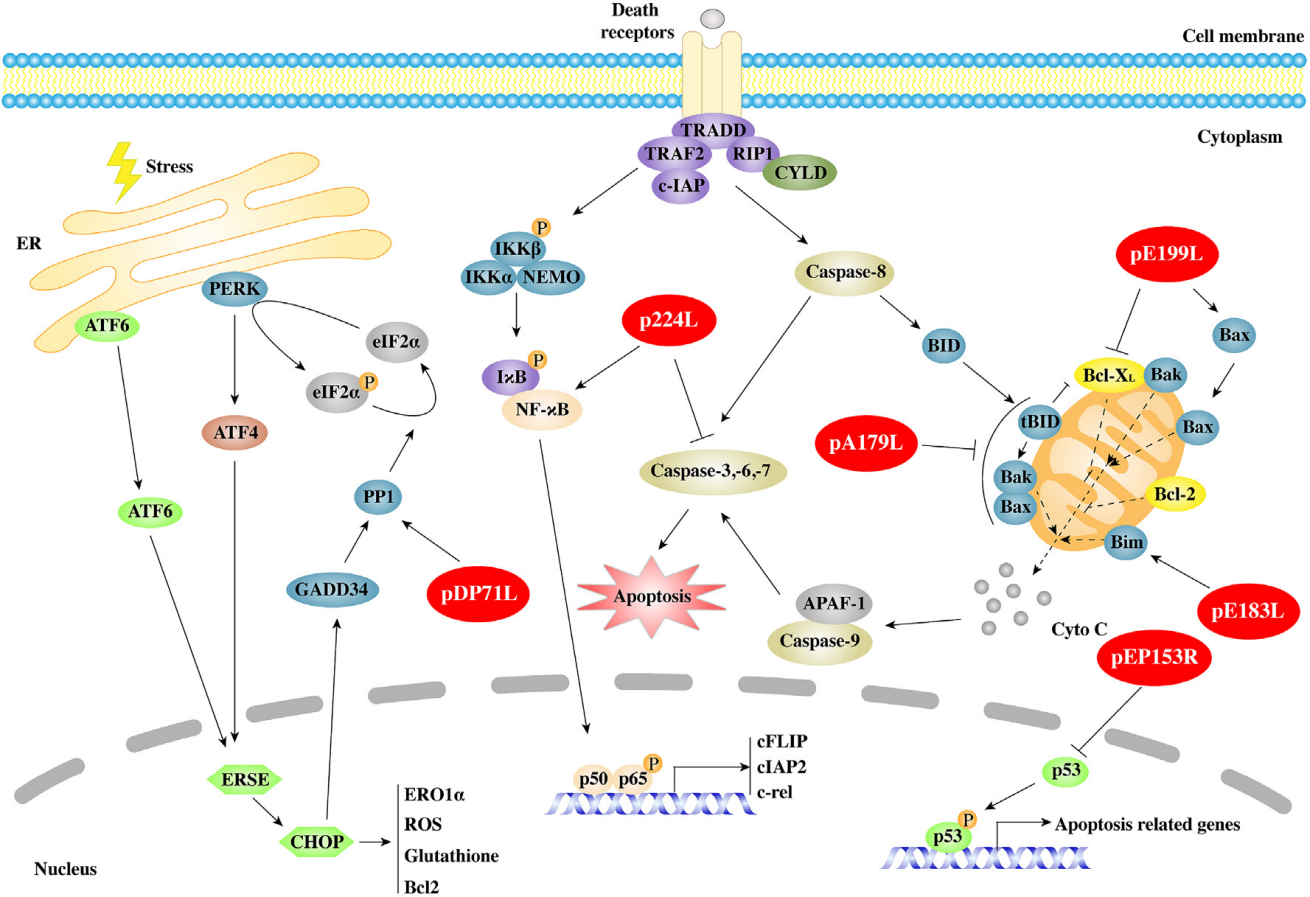
Modulating apoptosis by African swine fever virus (ASFV). ASFV proteins are shown in red ellipses. pA224L, an IAPs homolog protein, interacts with activated caspase-3 to inhibit apoptosis and elevates NF-κB activity to increase anti-apoptotic gene expressions of cFLIP, cIAP2 and c-rel. pA179L, a Bcl-2 homolog, interacts with a series of BH3-only proteins and prevents apoptosis. pDP71L interacts with eIF2α and then dephosphorylates eIF2α by recruiting PP1α, thus suppressing eIF2α-ATF4-CHOP apoptotic pathway. pEP153R reduces the transactivating activity of the cellular protein p53 and then inhibits apoptosis related gene expressions. pE183L (p54) induces apoptosis via possibly pro-apoptotic factor Bim translocation to mitochondria. pE199L competes with Bak for anti-apoptotic factor Bcl-X_L_ to activate pro-apoptotic factor Bak, and promotes another pro-apoptotic factor Bax translocation to mitochondria, thus inducing mitochondrial dependent apoptosis.

Induction of apoptosis of infected macrophages *in vivo* is one of the markers of acute ASF (Ramiro-Ibanez et al., 1997; Dixon et al., 2017). As ASFV replicates in large numbers, viral proteins are translated and modified in the endoplasmic reticulum, inducing endoplasmic reticulum stress response, and activating apoptosis signal pathway through caspase-12, ATF6 and other proteins (Dixon et al., 2017). In addition, mitochondria are actively recruited to the periphery of virus factory in a microtubule dependent manner. Morphological analysis shows that the recruited mitochondria actively provide ATP for ASFV replication and assembly (Rojo et al., 1998; Cobbold et al., 2000). Alternatively, mitochondrial recruitment may be a part of antiviral response involving mitochondrial driven apoptosis (Netherton and Wileman, 2013). Therefore, in order to facilitate virus replication and immune evasion, ASFV encodes some proteins to delay apoptosis. Interestingly, since ASFV can be transmitted by phagocytosis of apoptotic bodies by adjacent macrophages, promoting apoptosis may be advantageous for viruses to spread by increasing virus release from the cell and avoiding the induction of inflammatory signals (Vallee et al., 2001; Dixon et al., 2017). This suggests that some ASFV proteins activate cell apoptosis signaling pathways. Next, we will discuss the different roles of ASFV proteins in apoptosis and their molecular mechanism (Fig. 3).

#### 2.3.1 pA224L, a IAP homolog protein

The inhibitor of apoptosis protein (IAP) family is able to inhibit apoptosis in cells induced by many apoptosis triggering factors. It is well known that human IAPs bind to caspase-3 and caspase-7 and strongly suppress their protease activity. In addition, they can also inhibit the proteolysis of procaspase-3, procaspase-6, and procaspase-7 via blocking caspase-9 activated by cytochrome C. IAPs share similar structural characteristics, including 1–3 baculoviral IAP repeat (BIR) motifs and RING fingers. BIRs take part in the interaction between IAPs and other proteins, which are necessary for the anti-apoptosis activity of IAPs. RING fingers mediate ubiquitin ligase activity, which is required for the ubiquitinylation of the RIPK1 complex (Nogal et al., 2001; Dixon et al., 2017).

pA224L expressed in the advanced stage of ASFV infection, contains 224 amino acids and is relatively conserved in different ASFV strains, sharing 90%–99% amino acid identity. pA224L is a member of IAPs family, and sequence alignment shows that pA224L has a BIR motif at N-terminus and a predicted RING domain instead of canonical RING motif at C-terminus (Dixon et al., 2017; Wang J. et al., 2020). It has been shown that overexpression of pA224L significantly disturbs apoptosis induced by TNFα, cycloheximide or staurosporine in Vero cells. Further study indicates that pA224L is able to interact with the activated caspase-3 and inhibit its function. Deletion mutant of ASFV lacking *A224L* gene exhibits an increased ability to induce apoptosis compared with the wild type (Nogal et al., 2001). It is suggested that there are some alternative functions for pA224L to modulate host immune response including apoptosis. Transient transfection of *A224L* gene elevates NF-κB activity via increasing the activity of IKKs (Rodriguez et al., 2002). pA224L also controls the transcription of the translation initiation factor eIF4F through targeting Myc/Max/Mad network (Sanchez et al., 2013). Interestingly, deletion of *A224L* does not reduce the virulence of Malawi Lil-20/1 strain in pigs (Neilan et al., 1997), while immunization with *A224L* deletion mutant from NH/P68 strain fully protects pigs against homologous strain L60 challenges (Gallardo et al., 2018).

#### 2.3.2 pA179L, a B-cell lymphoma-2 (Bcl-2) homologue

Several apoptosis regulators belong to Bcl-2 family, including proapoptotic and antiapoptotic factors. Bcl-2 family has four Bcl-2 homology regions (BH1–BH4). The proapoptotic factor BH3-only proteins, including Bid, Bad, Bim, Bik, etc, can sense cellular damage and initiate the death process. Bax and Bak act as downstream of BH3-only proteins to change the permeability of mitochondrial membrane and result in the release of cytochrome C, which mediates the activation of the caspase cascade to cause apoptosis (Petros et al., 2004).

pA179L is expressed throughout the virus infection cycle, with a molecular mass of 21 ​kDa and 179 amino acids. It is highly conserved in different ASFV strains and shares 94%–99% identity in amino acids. pA179L is supposed to localize in mitochondria or endoplasmic reticulum (Hernaez et al., 2013). Early analysis demonstrates that pA179L as a member of Bcl-2 family contains conservative domains of BH1, BH2, BH3 and BH4, but lacks the corresponding transmembrane domains (Afonso et al., 1996). It not only prevents strong apoptosis activated by p68 kinase in HeLa and BSC-40 ​cells and by macromolecular synthesis in the human myeloid leukemia cell line K562, but also extends the survival of monolayer growing insect cells rather than suspension cultured cells (Brun et al., 1996, 1998; Revilla et al., 1997). These observations suggest that pA179L has low species specificity and may play a role in both *Sus scrofa* and *Ornithodorus* genera. Through multiple protein-protein interaction experiments, it is shown that pA179L directly interacts with a series of BH3-only proteins, including active truncated Bid, and other proapoptotic factors such as Bax and Bak, and then prevents cells from entering the apoptotic program (Galindo et al., 2008; Banjara et al., 2017). In addition to apoptosis, pA179L is also able to interact with Beclin-1, an autophagy-related protein, via its BH3 domain, and inhibits autophagy induced by starvation conditions (Hernaez et al., 2013).

#### 2.3.3 pDP71L, a translation regulatory protein

Virus infection causes ER stress in many ways, including aggregation of unfolded proteins in the ER, and then induces cell death. It has been proved that three ER transmembrane proteins are able to sense and counterbalance ER stress through distinct signaling pathways (Todd et al., 2008). PKR-like endoplasmic reticulum kinase (PERK) activated by ER stress has the capability to phosphorylate translation initiation factor eIF2α at Ser51. eIF2α forms a ternary complex with GTP and initiator Met-tRNA, which is necessary for eIF2α to recognize the initiation codon and initiate translation. Phosphorylation of eIF2α (p-eIF2α) influences the exchange of eIF2α-GDP and reduces global protein synthesis due to the increased affinity between p-eIF2α and the guanine nucleotide-exchange factor eIF2B (Harding et al., 1999; Krishnamoorthy et al., 2001). Paradoxically, p-eIF2α can increase the translation of several genes such as ATF4 (activating transcription factor 4) and CHOP (pro-apoptotic CCAAT enhancer binding protein homologous protein). CHOP, as a downstream target of ATF4, down-regulates Bcl2 transcription, depletes cellular glutathione, exaggerates production of ROS and induces ER oxidase 1α (ERO1α), thus resulting in apoptosis (McCullough et al., 2001; Zhang et al., 2010).

pDP71L, encoded by *DP71L* gene, is a highly conserved late protein in ASFV. Among different strains, pDP71L exists as either a long form (also named 23-NL, 184 amino acids) or a short form (also named NL-S, 70–72 amino acids). The NL-S protein shares 94%–100% amino acid identity with each other. The 23-NL shares 61% identity with NL-S on amino acid sequence 7–71 in NL-S and 118–185 in 23-NL (Zsak et al., 1996; Zhang et al., 2010; Dixon et al., 2017). Early studies have suggested that pDP71L is a virulence factor of the virus, and shares significant similarity with Myd116, GADD34 (host DNA damage inducible protein), and ICP34.5 (a neurovirulence-associated gene of HSV-1). Both GADD34 and ICP34.5 can recruit protein phosphatase 1 catalytic subunit (PP1α) to dephosphorylate eIF2α, and therefore restore global translation (Brush et al., 2003; Cheng et al., 2003). As expected, pDP71L is demonstrated to bind PP1α by yeast two-hybrid screening (Rivera et al., 2007). Subsequent studies have shown that pDP71L interacts with eIF2α and then dephosphorylates eIF2α by recruiting PP1α, thus promoting translation and suppressing eIF2α-ATF4-CHOP apoptosis pathway (Zhang et al., 2010). Based on years of research, it is believed that pDP71L is required for virulence in some ASFV strains. Deletion of *DP71L* from E70 and Georgia 2007 strains effectively reduces virus virulence *in vivo*, while the same deletion from Malawi Lil20/1 and Pretoriuskop/96/4 strains only slightly decreases virulence (Zsak et al., 1996; Afonso et al., 1998; Ramirez-Medina et al., 2019). Notably, induction of CHOP is still affected in cells infected with ASFV strain lacking *DP71L* gene. These data suggest that ASFV may encode other proteins that can compensate for the loss of pDP71L.

#### 2.3.4 pEP153R, a C-type lectin domain containing protein

*EP153R* has two different transcription initiation sites, one for late transcription close to the translation initiation codon, and one for early transcription at 164 nucleotide from the translation initiation codon. Thus, transcription of *EP153R* takes place at both early and late stages during the virus infection (Galindo et al., 2000). Analysis of *EP153R* gene of ASFV strain BA71V has predicted that pEP153R contains a C-type lectin-like region, a central transmembrane region, and a cell attachment (RGD) sequence. Deletion of *EP153R* from ASFV BA71V strain does not reduce virus virulence *in vitro*, but inhibits the hemadsorption phenomenon induced in ASFV-infected cells (Yanez et al., 1995; Galindo et al., 2000). Later studies have proved that Δ*EP153R* strain activates more caspase-3 and induces apoptosis in cells compared with its parental BA71V strain. Expression of pEP153R in Vero or COS cells results in partial inhibition of apoptosis induced by actinomycin, staurosporine, or ASFV. Further experiments have shown that pEP153R reduces the transactivating activity of the cellular protein p53, which is a cellular component of the apoptotic cascade (Hurtado et al., 2004).

#### 2.3.5 p54, a proapoptotic factor

ASFV protein p54, a late virus protein encoded by *E183L* gene, is located at the inner envelope of the mature virions (Rodríguez et al., 2004). As a structural protein, p54 is essential for virus attachment and replication (Rodriguez et al., 1996; Gomez-Puertas et al., 1998). Moreover, p54 directly binds to the light chain of cytoplasmic dynein (DLC8) and ensures efficient virion transporting in cells. Mapping of DLC8 binding region in p54 finds that amino acid residues at positions 149 to 161 are sufficient for its binding to DLC8, and an SQT motif (159–161) within this region is essential for this binding (Alonso et al., 2001). In a follow-up study, the amino-acid sequence required for p54-DLC8 interaction is the same with the binding domain of Bim, a member of the pro-apoptotic BH3-only protein. Therefore, p54 may play a role in the regulation of apoptosis by displacement of Bim from microtubules to mitochondrion. As expected, expression of p54 results in caspase-3 activation and cell apoptosis. Meanwhile, the 13-amino-acid domain of p54 required for DLC8 binding is also necessary for caspase-3 activation (Hernaez et al., 2004). Although p54 can be packed in virions, due to the existence of apoptosis inhibitor protein such as pA179L, p54 fails to effectively induce apoptosis in the early stage of infection. At the later stage of virus infection, high expression of p54 may induce apoptosis and promote virus transmission.

#### 2.3.6 pE199L, a viral inner-envelope protein

pE199L with molecular mass 19–20 ​kDa, also called j18L, is expressed at late times after ASFV infection and localized in viral inner envelop (Sun et al., 1996; Matamoros et al., 2020). Its amino acid sequence is highly conserved among different ASFV strains. pE199L is a type I transmembrane protein, containing a N-terminal cysteine-rich long segment, a potential transmembrane domain and a short C-terminal tail (Matamoros et al., 2020). Biological function analysis shows that pE199L is not required for ASFV morphogenesis, attachment, endocytosis and egress, but is critical for inner membrane fusion and genome-containing core penetration into the cytoplasm (Matamoros et al., 2020). Previous article suggests that pE199L interacts with and down-regulates pyrroline-5-carboxylate reductase 2 (PYCR2) to induce complete autophagy in Vero and HEK293T cells (Chen et al., 2021). Recently, pE199L is identified as an apoptosis inducer. Further detail analysis shows that pE199L competes with Bak for anti-apoptotic factor Bcl-X_L_ to activate pro-apoptotic factor Bak. In addition, pE199L also promotes the activation of another pro-apoptotic factor Bax and its translocation to mitochondria, leading to mitochondrial dependent apoptosis (Li et al., 2021).

## 3 Escape from adaptive immunity

It is well known that ASFV infection cannot induce adaptive immune response commendably. However, studies have shown that protective antibodies can be produced in pigs immunized with attenuated ASFV strains, and a large number of neutralizing antibodies can also be produced in pigs that survive the infection (Sanchez et al., 2019). The antibody adoptive transfer experiment has confirmed that the sera of pigs immunized with the attenuated strain could improve the resistance of healthy pigs to the homologous virulent strain (Onisk et al., 1994). In addition, piglets born to sows survived after ASFV infection can resist the infection of ASFV during lactation. These results imply that antibodies play an important role in hosts against ASFV infection (Schlafer et al., 1984a, 1984b). At the same time, T cells, NK cells, and NKT cells will be activated in pigs immunized with the attenuated strains, which will improve the resistance of pigs to the homologous virulent strains (Wang et al., 2020).

These observations suggest that adaptive immunity plays an important role in the resistance to ASFV infection. However, due to the high pathogenicity of ASFV and the interference of innate immunity, most of the infected pigs will die before they can produce sufficient immune protection. In the process of ASFV infection, some ASFV proteins might play roles in impairing host adaptive immunity.

### 3.1 pEP402R, an ASFV adhesion protein

pEP402R (also called CD2v, CD2-Like, 5HL and 8-DR) is a late expressed protein and integrated into outer envelope of ASFV virions. The structure and function of pEP402R is similar to the T-lymphocyte surface antigen CD2 expressed on T cells and NK cells. pEP402R mediates the adhesion of extracellular virus to red blood cells, and promotes the spread of virus in hosts. Deletion of *EP402R* from the BA71 virulent strain results in virus attenuation, and the attenuated virus can induce protection against the parental virulent virus (Monteagudo et al., 2017). However, deletion of *EP402R* from Malawi Lil-20/1 strain does not reduce its virulence, although a delay on the onset of clinical signs and virus dissemination is observed (Borca et al., 1998). In addition, pEP402R can inhibit mitogen-dependent lymphocyte proliferation to suppress adaptive immunity, which might be mediated by the direct interaction between pEP402R and lymphocyte surface ligand, or by the interaction between pEP402R and cytoplasmic proteins (Borca et al., 1998; Dixon et al., 2004).

A previous study has shown that pEP402R protein interacts with the adaptor protein AP-1 (Perez-Nunez et al., 2015). AP-1 is a cytosolic heterotetramer involved in the transport of protein cargo from the trans-Golgi network (TGN) to endosomes. Binding to AP-1 might be involved in MHC-I (class I major histocompatibility complex) down-regulation and cross-presentation (Wonderlich et al., 2008; Perez-Nunez et al., 2015). Besides, pEP402R contains variable numbers of a proline rich repeat, which binds to SH3P7/mAbp1, an actin-binding adaptor protein (Kay-Jackson et al., 2004). These results indicate that pEP402R may modulate vesicular transport and signal transduction.

### 3.2 Other ASFV proteins involved in adaptive immunosuppression

3D structure simulation of pEP153R predicts that the dimer of pEP153R may interact with MHC-I. iPAM cells infected with ASFV Δ*EP153R* express more MHC-I on cell surface. Stable and transient expression of pEP153R in iPAM or Jurkat cells proves that pEP153R inhibits the expression of MHC-I on cell membrane, most likely by destroying the exocytosis process without affecting the synthesis and glycosylation of MHC-I (Hurtado et al., 2011). pEP153R is homologous to several cellular proteins such as CD94, CD69, Ly94A, and CD44. CD44 is involved in cellular adhesion and T-cell activation (Galindo et al., 2000; Hurtado et al., 2011).These data suggest that pE153R regulates the adaptive immune response induced by ASFV infection. As mentioned above, pA238L and pA224L may also affect the activation of T cells or other immune cells through the NFAT pathway or NF-κB pathway.

## 4 Conclusions

Since ASFV re-emerged in the Caucasus region in 2007, the disease has spread rapidly worldwide and caused great economic impact on swine industry. It is believed that the failure of host innate and adaptive immune responses to control virus replication is the key factor leading to the rapid ASFV replication and high mortality in infected pigs. At present, progresses have been made in understanding the interaction between ASFV and host innate and adaptive immune responses, but more mystery remains to be investigated. Evidence suggests that the inhibition of IFN-I production and function by some ASFV proteins is of critical importance in facilitating rapid virus replication. Knockdown or deletion of coding genes of these proteins can attenuate the virus in some ASFV strains but not affect the virulence in other ASFV strains. The reason could be that ASFV has complex gene structures and encodes a large number of virus proteins, which have redundant functions. Therefore, it is necessary to further explore the function and detailed molecular mechanisms of ASFV proteins in the future. In addition, different genotypes of ASFV may also be the reason. Thus, the genetic background of the strains must be taken into account in the follow-up studies on the functions of ASFV proteins. This will provide more references for vaccine development.

The delayed onset of apoptosis in infected cells and induction of apoptosis in bystander lymphocytes are key factors in enabling ASFV replication and immune evasion. However, how ASFV achieves a balance between inhibiting and promoting apoptosis needs to be further studied. Moreover, pyroptosis and necroptosis are both a form of cell death pathways and are conducive to host virus clearance. A better understanding of the impact of ASFV on cell death pathways will help us understand ASFV pathogenesis and develop effective vaccines.

## Conflict of interest

The authors declare that they have no conflict of interest.
